# Supplementary material for: Multi-Scale Feature Attention Network for Rapid and Non-Destructive Quantification of Aflatoxin B1 in Maize Using Hyperspectral Imaging
Source: Foods. 2025 Nov 3;14(21):3769. doi: 10.3390/foods14213769 (PMC12610444; doi:10.3390/foods14213769)
Supplement: Supplementary file 1 [file foods-14-03769-s001.zip › foods-3946862-supplementary.pdf]

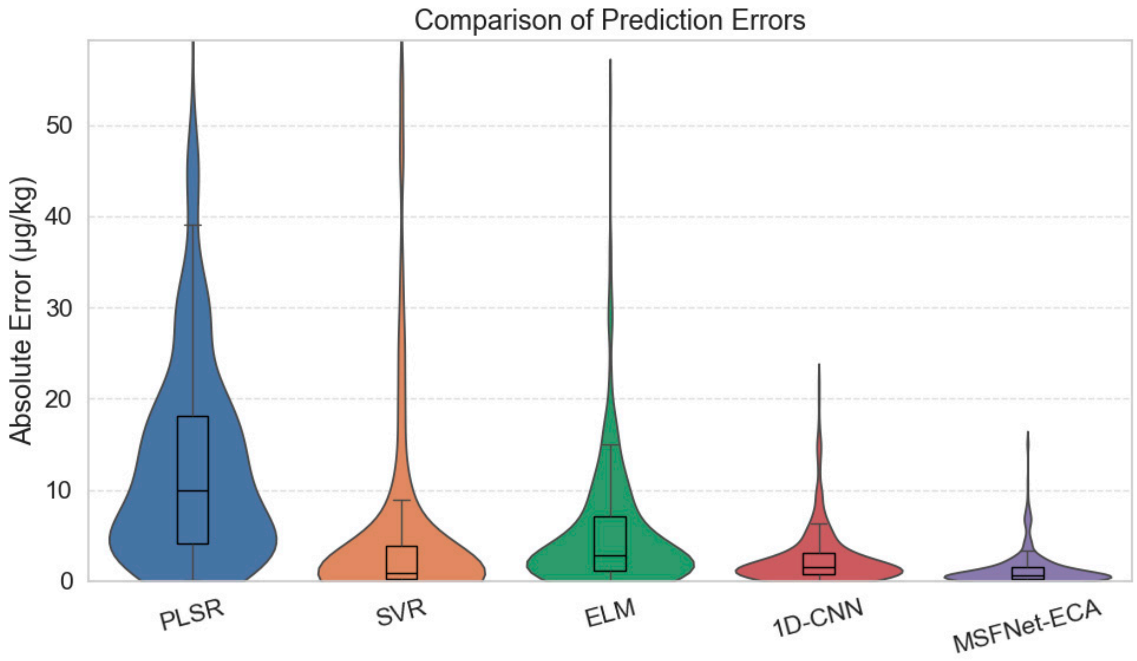

Figure S1. Comparison of prediction errors for different models (PLSR, SVR, ELM, 1D-CNN, and MSFNet-ECA). The violin plots illustrate the distributions of absolute prediction errors across the SPXY test set, showing that the MSFNet-ECA model achieved the lowest and most stable errors compared with other methods.
